# Tumor necrosis factor-α −308 G/A polymorphism and risk of sepsis, septic shock, and mortality: an updated meta-analysis

**DOI:** 10.18632/oncotarget.20862

**Published:** 2017-09-13

**Authors:** Hao Wang, Shujin Guo, Chun Wan, Ting Yang, Ni Zeng, Yanqiu Wu, Lei Chen, Yongchun Shen, Fuqiang Wen

**Affiliations:** ^1^ Department of Respiratory and Critical Care Medicine, West China Hospital of Sichuan University and Division of Pulmonary Diseases, State Key Laboratory of Biotherapy of China, Chengdu 610041, China; ^2^ Internal Medicine of Sichuan Provincial People's Hospital and Sichuan Academy of Medical Science, Chengdu 610072, China

**Keywords:** sepsis, mortality, polymorphism, meta-analysis

## Abstract

**Background:**

The -308G/A polymorphism in the gene encoding tumor necrosis factor-α (TNF-α) has been implicated in sepsis risk in many studies but with variable results. This study aimed to comprehensively assess the evidence of association between this polymorphism and risk of sepsis and sepsis-related mortality.

**Materials and Methods:**

PubMed, EMBASE and other databases were searched to identify relevant studies, and data were analyzed using Review Manager 5.0 and STATA 12.0.

**Results:**

Data from 34 publications involving 12,284 subjects were meta-analyzed. Combined analysis revealed an association between TNF-α -308G/A gene polymorphism and risk of sepsis (AA+GA vs. GG, OR 1.35, 95% CI 1.10–1.67, *P* = 0.005). This association was observed in the Caucasian subgroup (OR 1.50, 95% CI 1.13–2.00, *P =* 0.006), but not in the Asian subgroup. Across the entire study population, the polymorphism was also significantly related to septic shock risk (OR 1.52, 95% CI 1.18–1.95, *P =* 0.001) but not to sepsis-related mortality (OR 0.99, 95% CI 0.71–1.40, *P =* 0.97).

**Conclusions:**

This meta-analysis suggests that the -308G/A gene polymorphism in the TNF-α gene may contribute to risk of sepsis and septic shock, but not risk of mortality.

## INTRODUCTION

Sepsis, which is usually caused by infection and mediated by severe systemic inflammation, creates a heavy disease burden worldwide. It is more likely than any other infectious disease to impair quality of life and ability to work. Despite extensive progress in sepsis management in recent decades, sepsis-related mortality remains as high as 30–50% [1]. This may reflect the lack of specific therapeutic strategies, which has inspired a growing literature aimed at investigating the pathogenesis of sepsis in order to explore unique therapeutic targets.

Growing evidence indicates that inflammatory cytokines play vital roles in regulating the host immune response and amplifying inflammation in sepsis. Single-nucleotide polymorphisms (SNPs) in the genes encoding these cytokines may be related to the risk of sepsis, and may even play roles in its pathogenesis [2, 3]. Among these diverse cytokines, tumor necrosis factor-α (TNF-α) has attracted considerable attention. TNF-α is an intensively studied pro-inflammatory cytokine released mainly by activated neutrophils and macrophages that helps regulate the mammalian immune response and cellular homeostasis [4]. It stimulates various immune and structural cells, triggering release of large amounts of inflammatory mediators, which amplify the inflammatory response and cause severe systemic inflammation, eventually leading to sepsis [5].

Numerous studies have examined various SNPs in the TNF-α gene and their potential relationship to sepsis risk. In particular, several studies of the −308G/A polymorphism have suggested a relationship, but others have given contrasting results [6–9]. A systematic review in 2010 concluded that this polymorphism is associated with risk of sepsis, but not risk of sepsis-related mortality [10]. Because several studies on this potential association have been published since that review, we wanted to perform an updated meta-analysis in order to gain a comprehensive understanding of all available evidence through the end of 2016, including the studies analyzed in the 2010 review. We examined the potential associations of this polymorphism with risk of sepsis, septic shock and sepsis-related mortality.

## RESULTS

### Characteristics of included publications and studies

After systematic search of the literature and study selection, 34 publications (37 studies) involving 12,284 subjects from 16 countries were included in the meta-analysis (Table 1 and Supplementary Figure 1) [11–44]. These studies were published between 1999 and 2015. Fourteen studies were performed in Caucasians [22, 25–26, 28–30, 32–34, 36–37, 40–41, 43], 12 in Asians [11, 13–16, 19–20, 24, 31, 38–39, 44], and the remainder in populations of mixed or unknown ethnicity [12, 17–18, 21, 23, 27, 35, 42]. In contrast, the most recent systematic review and meta-analysis of associations between the −308G/A polymorphism and sepsis contained 25 studies involving 2,977 patients [10]. Studies incorporated since that last review are marked with an asterisk in Table 1.

**Table 1:** Clinical summary of included studies

Author/Year	Country	Ethnicity	Age group	Patient group	Sepsis type	Subjects	SNP method	Sepsis risk reported	Septic shock risk reported	Mortality reported
Allam et al. 2015*	Saudi Arabia	Asian	Neonate	NICU	S	137	Taqman	Y	N	N
Cardoso et al. 2015*	Brazil	Mixed	≥ 18	ICU	S,SS, SSH	72	Taqman	N	N	Y
Feng et al. 2015*	China	Asian	NA	Pneumonia	SS, SSH	277	Taqman	N	N	Y
Gupta et al. 2015*	India	Asian	≥ 16	Trauma	S	114	PCR-SSP	Y	N	N
Baghel et al. 2014*	India	Asian	≥ 18	Post-operative	S	239	PCR	Y	N	N
Kothari et al. 2013*	India	Asian	NA	Critical ill patient	S, SSH	400	PCR	Y	Y	N
Susantitaphong et al. 2013*	USA	Mixed	≥ 18	AKI	S	262	PCR	Y	N	N
Azevedo et al. 2012*	Brazil	Mixed	< 18	PICU patients	S,SS, SSH	1003	Taqman	Y	N	Y
Song et al. 2012*	China	Asian	NA	Trauma and Critical ill patient	S,SS	1402	PCR	Y	N	Y
Duan et al. 2011*	China	Asian	≥ 18	Trauma	S	305	PCR-RFLP	Y	N	N
Härtel et al. 2011*	Germany	Mixed	Infant	VLBWI	S	2870	PCR	Y	N	N
Paskulin et al. 2011*	Brazil	Caucasian	≥ 18	Critical ill patient	S, SSH	520	PCR-RFLP	Y	Y	Y
Carregaro et al. 2010 *	Brazil	Mixed	≥ 18	ICU patients	S,SS, SSH	303	Taqman	Y	N	N
Gu et al. 2010*	China	Asian	≥ 18	Trauma	S	305	PCR	Y	N	N
Menges et al. 2008	Germany	Caucasian	≥ 18	Trauma	S	230	PCR	Y	N	N
Jessen et al. 2007	Denmark	Caucasian	≥ 17	Gram negative	S,SS, SSH	304	PCR	N	N	Y
McDaniel et al. 2007	USA	Mixed	NA	Trauma	S	68	PCR	Y	N	N
Garnacho-Montero et al. 2006	Spain	Caucasian	> 18	ICU	S,SS, SSH	325	PCR	Y	Y	Y
Schueller et al. 2006	Germany	Caucasian	< 32 weeks	Premature infant	S	169	PCR	Y	N	N
Sipahi et al. 2006	Turkey	Caucasian	< 15	Critical ill patient	SS	130	PCR	Y	N	Y
Nakada et al. 2005	Japan	Asian	NA	Critical ill patient	S	411	PCR-RFLP	Y	N	Y
Gordon et al. 2004	UK and Australia	Caucasian	≥ 18	ICU patient	SS, SSH	566	PCR-RFLP, PCR-SSP	Y	N	Y
Jaber et al. 2004	USA	Caucasian	≥ 18	ARF	S	61	PCR-SSP	Y	N	N
Balding et al. 2003	Ireland	Caucasian	< 16	Meningococaemia	S	572	PCR	Y	N	Y
Calvano et al. 2003	Spain	Mixed	> 21	Post-operative	S, SSH	44	PCR	Y	Y	Y
Schaaf et al. 2003	Germany	Caucasian	NA	Pneumococcal infection	S,SS, SSH	118	PCR	Y	Y	Y
Treszl et al. 2003	Hungary	Caucasian	Infant	LBWI	S	103	PCR-RFLP	Y	N	N
Zhang et al. 2003	China	Asian	NA	ASP	SSH	148	PCR	Y	Y	N
Zhang et al. 2003	China	Asian	NA	ABP	SSH	120	PCR	Y	Y	N
Majetschak et al. 2002	Netherlands	Caucasian	≥ 18	Trauma	SS	70	PCR	Y	N	N
Appoloni et al. 2001	Belgium	Caucasian	NA	ICU patient	SSH	34	PCR	N	N	Y
Waterer et al. 2001	Australia	Mixed	NA	CAP	S	280	PCR	Y	N	Y
Mira et al. 1999	France	Caucasian	NA	ICU patient	SSH	176	PCR	Y	Y	Y
Nuntayanuwat et al. 1999	Thailand	Asian	NA	Meliodosis	S	146	PCR-RFLP	Y	N	N

ABP: Acute biliary pancreatitis; AKI: Acute kidney injury; ARF: Acute renal failure; ASP: Acute severe pancreatitis; CAP: Community acquired pneumonia; ICU : Intensive care unit; LBWI: Low birth weight infant; NA: Not available; PCR: Polymerase Chain Reaction; PCR-RFLP: Polymerase chain reaction-restriction fragment length polymorphism; PCR-SSP: Polymerase chain reaction -sequence-specific amplification; PICU: Pediatric intensive care unit; S: Sepsis; SS: Severe sepsis; SSH: Septic shock; N: No; Y: Yes; VLBWI: Very Low birth weight infant; *: Studies which were newly included in the review when compare with previous meta-analysis.

Of the 34 publications, 30 (33 studies) examined correlations between the TNF-α −308 A/G polymorphism and risk of sepsis [11, 14–25, 27–40, 42–44], 8 examined correlations between this polymorphism and risk of septic shock [16, 22, 28, 35–36, 38–39, 43], and 16 examined correlations between this polymorphism and risk of sepsis-related mortality [12–13, 18–19, 22, 26, 28, 30–32, 34–36, 41–43]. Genotype distributions in case and control groups are shown in Supplementary Table 1. Distributions in control groups met the criteria of Hardy-Weinberg equilibrium (HWE) in 24 publications (71%).

Study quality is assessed in Supplementary Table 2. Most publications (26 of 34, 76%) provided detailed information about genotyping primers, and 32 publications reported the definition of sepsis, usually based on the definitions recommended by the American College of Chest Physicians/Society of Critical Care Medicine or by the International Sepsis Definitions Conferences [45–46]. On the other hand, fewer than one-third of publications (10 of 34, 29%) reported procedures for blinding.

### TNF-α −308 A/G polymorphism and sepsis risk

**Figure 1:**
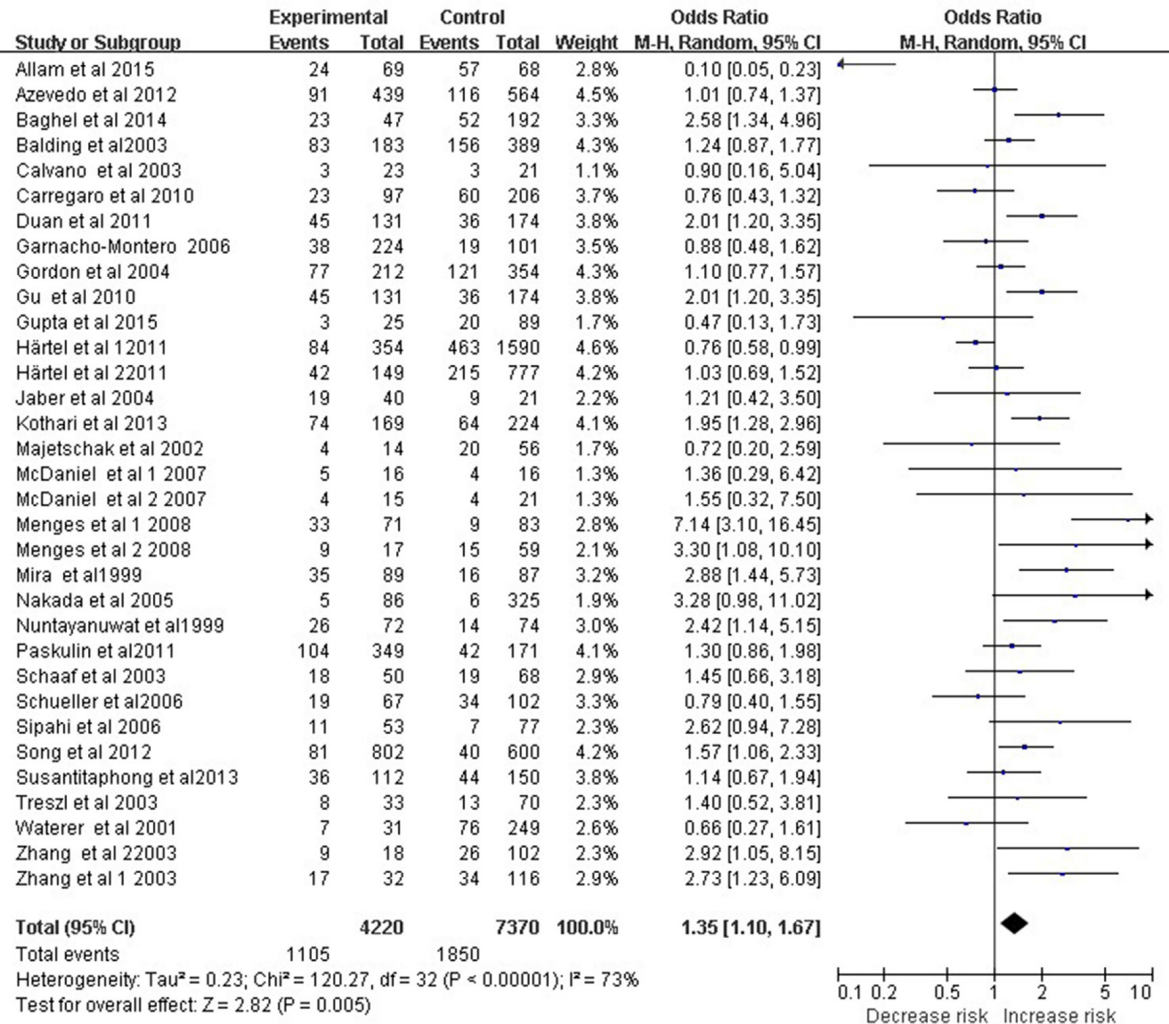
Meta-analysis to evaluate the association between the TNF-α -308 A/G polymorphism and sepsis risk (AA+GA vs. GG) The size of the square is proportional to the weight of each study; horizontal lines represent the 95% CI.

A total of 33 studies involving 11,590 subjects evaluated relationships between the TNF-α −308 A/G polymorphism and overall risk of sepsis, which included sepsis, severe sepsis, and septic shock. The dominant model (AA+GA *vs.* GG) indicated a significant association between the TNF-α −308 A/G polymorphism and sepsis risk (OR 1.35, 95% CI 1.10–1.67, *P* = 0.005; Figure 1). However, meta-analysis of only the 23 studies meeting HWE criteria showed no such association based on the dominant model (OR 1.28, 95% CI 0.99–1.64, *P* = 0.06).

**Figure 2:**
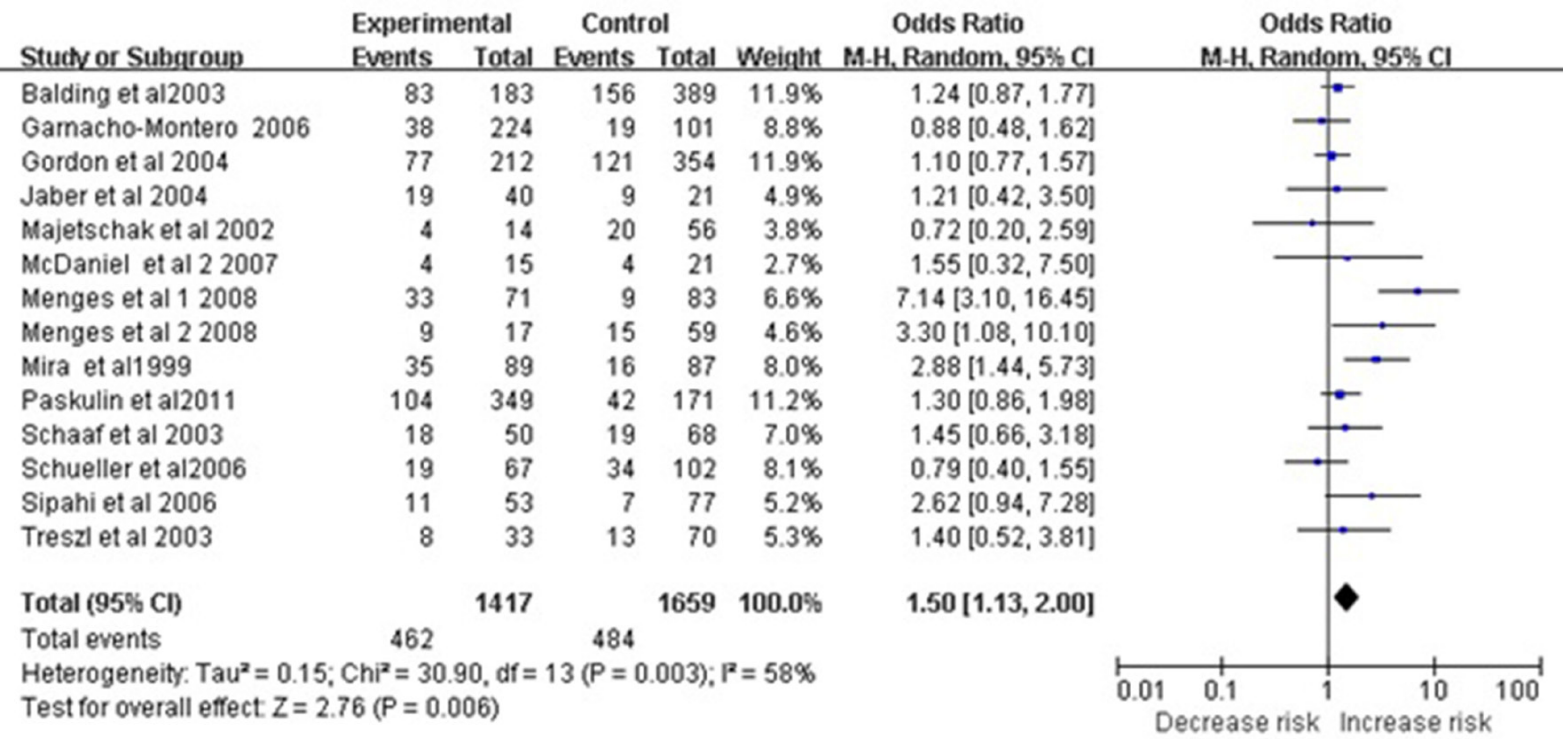
Meta-analysis to evaluate the association between the TNF-α -308 A/G polymorphism and sepsis risk in Caucasian (AA+GA vs. GG) The size of the square is proportional to the weight of each study; horizontal lines represent the 95% CI.

Applying the dominant model to the subgroup of Caucasian subjects revealed a significant association between the polymorphism and sepsis risk (OR 1.50, 95%CI 1.13–2.00, *P* = 0.006; Figure 2). However, this association was not observed in the subgroup of Asian subjects (OR 1.56, 95% CI 0.97–2.51, *P* = 0.07). Results of meta-analyses assessing relationships between the −308 A/G polymorphism and overall risk of sepsis are summarized in Table 2.

**Table 2:** Summary of statistical results

Statistical model	Group	Number of studies	*Q* test *P* value	I^2^	Model	OR (95% CI)	*P* value
AA+GA vs. GG	Overall Sepsis	33	*P* < 0.00001	73%	Random Model	1.35 [1.10, 1.67]	0.005
	Studies with HWE	23	*P* < 0.00001	78%	Random Model	1.28 [0.99, 1.64]	0.06
	Ethnicity						
	Caucasian	14	*P* = 0.003	58%	Random Model	1.50 [1.13, 2.00]	0.006
	Asian	11	*P* < 0.00001	83%	Random Model	1.56 [0.97, 2.51]	0.07
	Septic shock	8	*P* = 0.17	32%	Fixed Model	1.52 [1.18, 1.95]	0.001
	Mortality	16	*P* = 0.03	45%	Random Model	0.99 [0.71, 1.40]	0.97
AA vs. GA+GG	Overall Sepsis	25	*P* = 0.002	54%	Random Model	1.14 [0.74, 1.74]	0.55
	Studies with HWE	21	*P* = 0.006	53%	Random Model	1.09 [0.67, 1.75]	0.74
	Ethnicity						
	Caucasian	9	*P* = 0.58	0%	Fixed Model	1.01 [0.61, 1.66]	0.97
	Asian	9	*P* < 0.0001	78%	Random Model	1.57 [0.55, 4.49]	0.40
	Septic shock	4	*P* = 0.55	0%	Fixed Model	2.78 [1.20, 6.40]	0.02
	Mortality	12	*P* = 0.49	0%	Fixed Model	1.75 [0.97, 3.18]	0.06
AA vs GG	Overall Sepsis	25	*P* < 0.0001	66%	Random Model	1.13 [0.68, 1.86]	0.64
	Studies with HWE	21	*P* < 0.0001	67%	Random Model	1.07 [0.60, 1.91]	0.81
	Ethnicity						
	Caucasian	9	*P* = 0.56	0%	Fixed Model	1.06 [0.64, 1.74]	0.83
	Asian	9	*P* < 0.00001	85%	Random Model	1.56 [0.42, 5.75]	0.51
	Septic shock	4	*P* = 0.55	0%	Fixed Model	2.84 [1.22, 6.59]	0.02
	Mortality	12	*P* = 0.50	0%	Fixed Model	1.60 [0.88, 2.92]	0.12
A vs G	Overall Sepsis	27	*P* < 0.00001	77%	Random Model	1.17 [0.96, 1.42]	0.12
	Studies with HWE	21	*P* < 0.00001	79%	Random Model	1.13 [0.90, 1.41]	0.30
	Ethnicity						
	Caucasian	9	*P* = 0.59	0%	Fixed Model	1.13 [0.96, 1.32]	0.13
	Asian	11	*P* < 0.00001	87%	Random Model	1.46 [0.91, 2.32]	0.12
	Septic shock	4	*P* = 0.66	0%	Fixed Model	1.30 [0.97, 1.75]	0.08
	Mortality	12	*P* = 0.18	27%	Fixed Model	0.91 [0.72, 1.15]	0.42

### TNF-α −308 A/G polymorphism and septic shock risk

**Figure 3:**
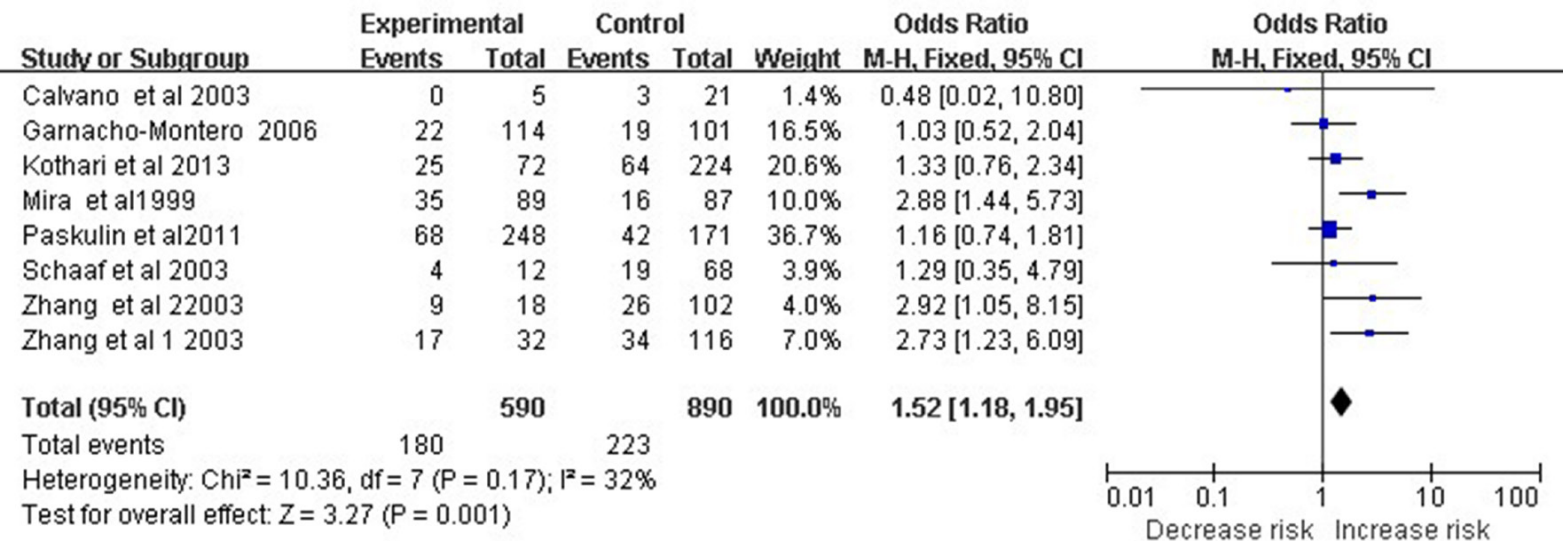
Meta-analysis to evaluate the association between the TNF-α -308 A/G polymorphism and septic shock risk (AA+GA vs. GG) The size of the square is proportional to the weight of each study; horizontal lines represent the 95% CI.

Eight studies involving 1,480 subjects examined association of the TNF-α −308 A/G polymorphism with septic shock risk. Meta-analysis revealed a significant association based on the dominant model (OR 1.52, 95% CI 1.18–1.95, *P* = 0.001; Figure 3).

### TNF-α −308 A/G polymorphism and sepsis-related mortality

A total of 16 studies involving 2,850 patients with sepsis, of whom 2,064 survived through the end of the study and 786 did not, examined the potential relationship between the TNF-α −308 A/G polymorphism and sepsis-related mortality. None of the four statistical models revealed significant differences in genotype frequencies at the TNF-α −308 A/G polymorphism between sur*vivo*rs and non-sur*vivo*rs (Table 2). Similar results were obtained for Caucasian and Asian subgroups (data not shown).

### Analysis of sensitivity and publication bias

**Figure 4:**
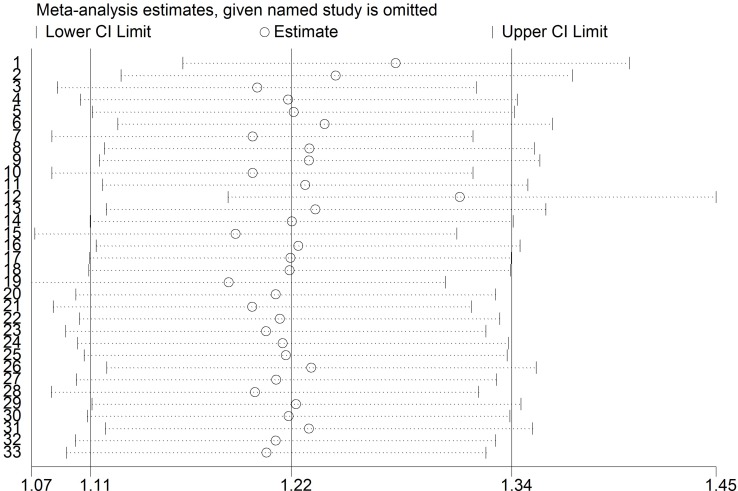
Sensitivity analysis of included studies examining the TNF-α promoter -308 A/G polymorphism and sepsis risk (CC vs. CG + GG)

**Figure 5:**
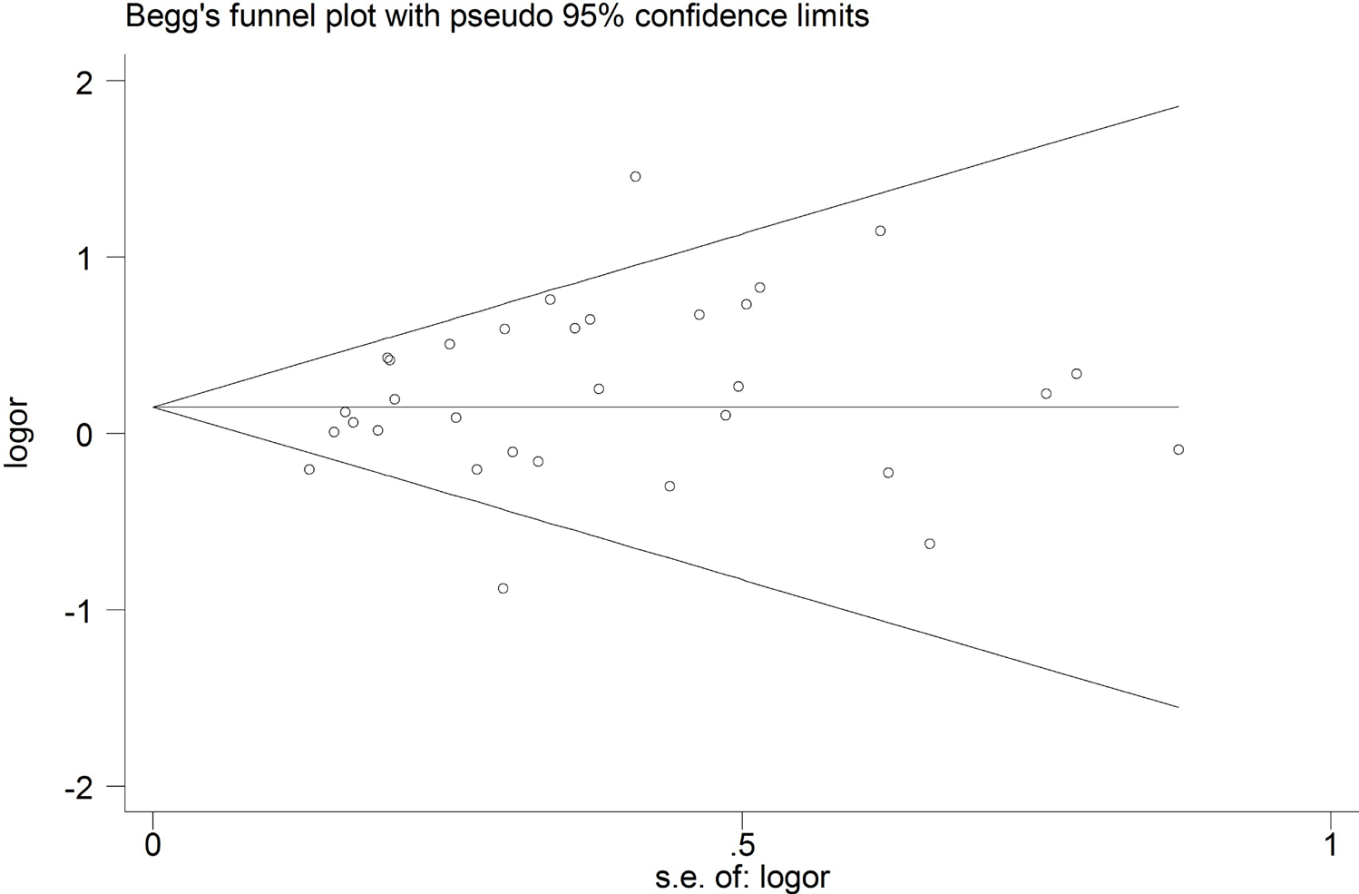
Begg’s funnel plot to detect publication bias in studies examining the TNF-α promoter -308 A/G polymorphism and sepsis risk (CC vs. CG + GG)

To assess the robustness of our meta-analysis results, we sequentially excluded each study from the complete set of 33 examining sepsis risk, and then we re-calculated ORs. In each case, the results were similar to those obtained with the complete set of studies (Figure 4). Begg’s funnel plot showed a symmetrical pattern for the dominant genetic model (AA+GA *vs.* GG) (Figure 5), and Egger’s test gave a *P* value of 0.63, suggesting a lack of publication bias.

## DISCUSSION

Our meta-analysis of the most complete and up-to-date set of studies published so far on potential relationships between the TNF-α −308 A/G polymorphism and sepsis suggests that this polymorphism significantly contributes to the risk of sepsis and septic shock, but not to risk of sepsis-related mortality. Thus, this polymorphism appears to be a risk factor for sepsis, but not a mortality predictor.

TNF-α, one of the most studied pro-inflammatory cytokines implicated in diverse diseases, is known to contribute to sepsis. The −308 A/G polymorphism in the TNF-α gene has been shown to up-regulate the gene’s transcription [47], ultimately leading to higher levels of expressed protein in serum [15–16, 19]. Elevated TNF-α levels enhance the inflammatory response and lead to the multiple phenotypic and functional characteristics of sepsis; they also induce apoptosis and reduce immune responsiveness and cell function [48–49].

An association between the −308 A/G polymorphism and sepsis risk was observed in the entire study population, as well as in the subgroup of Caucasian participants. However, this association was not observed in the subgroup of Asian participants. This may reflect different prevalence of the SNPs in different ethnic groups. It may also mean that genetic risk factors for sepsis differ across ethnic groups. Further research should examine the possibility of ethnic bias in the associations of sepsis risk with certain alleles/genotypes at the −308 A/G polymorphism in the TNF-α gene, with certain alleles/genotypes at other SNPs in the TNF-α gene, and with SNPs in other genes.

A subtype of sepsis is septic shock, which involves circulatory, cellular, and metabolic abnormalities. Septic shock is associated with higher risk of mortality than sepsis alone [50]. Our meta-analysis indicated a stronger association of the TNF-α −308 A/G polymorphism with risk of septic shock (OR 1.52, 95% CI 1.18–1.95) than with risk of sepsis (OR 1.35, 95% CI 1.10–1.67). However, the meta-analysis did not indicate a significant association between the polymorphism and sepsis-related mortality (OR 0.99, 95% CI 0.71–1.40). One possible explanation for the lack of association with mortality is that the SNPs in the TNF-α gene that influence protein expression differ between ethnic groups or even between individuals. Another explanation is environmental differences that may influence TNF-α transcription. It is also possible that, since other cytokines help to mediate sepsis, polymorphism in the TNF-α gene by itself is insufficient to significantly affect risk of sepsis-related death. Whether sepsis ultimately leads to mortality can also depend on numerous clinical factors, which cannot be predicted from genetics [51]. Our meta-analysis suggests that future research on risk factors of sepsis-related mortality may wish to focus on other SNPs in the TNF-α gene or on SNPs in other cytokine genes.

Our meta-analysis has several limitations. First, although we added several new studies relative the most recent meta-analysis from 2010 [10], the numbers of studies and subjects in our work were still limited. This may reduce the statistical power for identifying possible associations between TNF-α −308 A/G polymorphism and sepsis, especially septic shock, for which the meta-analysis included only 8 studies. Second, our meta-analysis included only studies in Asians, Caucasians, and populations of mixed or unknown ethnicity. Future work should examine this polymorphism in other populations, such as Hispanics, Africans, and Native Americans. Third, we reviewed only English-language publications. It is possible that our results would have been different if we had searched for literature in other languages. Fourth, we looked at only one SNP in only one sepsis-related gene. The available evidence, including results from the present meta-analysis, highlight the importance of taking into account the potential contributions of other cytokines and signaling pathways in sepsis risk.

Indeed, sepsis is a complicated clinical syndrome arising through interactions between genotype and environment [52]. Future studies should aim to avoid false associations resulting from ethnic differences in genotype frequencies and disease prevalence. Studies should also pursue high methodological quality, and genotype distributions in the controls should be confirmed to satisfy HWE. Functional studies that take into account genetic and environmental factors should investigate how the TNF-α −308 A/G polymorphism affects TNF-α production and the cytokine’s role in pathogenesis of sepsis. The results of this work may help improve risk assessment and precision treatment of patients with sepsis.

## MATERIALS AND METHODS

This meta-analysis was performed according to the guidelines of the Preferred Reporting Items for Systematic Reviews according to standard methods recommended for genetic meta-analysis [53–54]. Institutional review board approval was waived since this was a secondary data analysis.

### Literature search

Two authors (HW and SG) independently searched PubMed, EMBASE, Scopus, and Web of Science databases up to December 2016 in order to identify studies testing the association between the TNF-α −308 A/G polymorphism and sepsis risk. The following search terms were used: “Sepsis *or* severe sepsis *or* septic shock *or* septicemia *or* infection-related systemic inflammatory response syndrome” *and* “tumor necrosis factor-α *or* tumor necrosis factor-alpha *or* TNF-α” *and* “polymorphism *or* variant *or* mutation”. The reference lists of identified original studies and review articles were manually reviewed to find additional relevant publications.

### Study selection

Studies were included in the review if they (1) evaluated the association of the TNF-α −308 A/G polymorphism with risk of sepsis or septic shock or risk of sepsis-related mortality, (2) had a case-control design, (3) reported genotype distributions for case and control groups in a way that allowed estimation of odds ratios (ORs) and 95% confidence intervals (CIs), and (4) were published in English. When duplicate publications involved the same or overlapping data sets, only the study with the larger number of participants was included. If one article contained training and validation groups, each was treated as a separate study.

Abstracts and reviews were excluded from consideration, as were studies that failed to report genotype frequencies. Studies that included patients with sepsis caused by non-bacterial infection were excluded.

### Data extraction

Two authors (HW and SG) independently extracted data from the final set of included studies using a pre-designed table. The following data were collected: name of first author, year of publication, country of origin, ethnicity of subjects, source of subjects, sample size, sepsis type, genotyping method, and genotype frequencies in case and control groups. Any disagreement was resolved through discussion with the third author (CW).

### Assessment of study quality

The following questions were assessed for each study: (1) whether the genotype distribution in control groups was consistent with HWE; (2) whether the study listed primer sequences; (3) whether data analysts were blind to genotype and clinical status; and (4) whether a specific definition of sepsis was reported.

### Statistical analysis

Strength of the associations of the TNF-α −308 A/G polymorphism with risk of sepsis, septic shock and sepsis-related mortality was assessed using ORs and 95% CIs. Significance of the pooled OR was evaluated using the Z-test, with *P* < 0.05 defined as the significance threshold. Results were calculated according to four genetic models: dominant, AA+AG *vs.* GG; recessive, AA *vs.* AG+GG; additive, AA *vs.* GG; and allelic contrast, A *vs.* G. Subgroup analyses based on ethnicity were also performed.

Heterogeneity was evaluated using the χ^2^-based Q statistic as well as the I^2^ statistic, with *P* < 0.10 defined as the significance threshold. If *P ≥* 0.10, then data were meta-analyzed using a fixed-effects model; otherwise, a random-effects model was used. Publication bias was assessed using Begg’s funnel plots and Egger’s test [55–56]. Sensitivity analysis was carried out by sequentially excluding individual studies and re-calculating the ORs. All statistical tests were performed using Revman 5.0 (Cochrane Collaboration, Oxford, UK) and STATA 12.0 (Stata Corp., College Station, TX, USA).

## SUPPLEMENTARY MATERIALS FIGURE AND TABLES





## Abbreviations

TNF-α: tumor necrosis factor-α
OR: odds ratio
CI: confidence interval
SNP: single nucleotide polymorphism
HWE: Hardy-Weinberg equilibrium

## References

[1] Dellinger RP, Levy MM, Rhodes A, Annane D, Gerlach H, Opal SM, Sevransky JE, Sprung CL, Douglas IS, Jaeschke R, Osborn TM, Nunnally ME, Townsend SR (2013). Surviving Sepsis Campaign Guidelines Committee including the Pediatric Subgrou: Surviving sepsis campaign: international guidelines for management of severe sepsis and septic shock: 2012. Crit Care Med.

[2] Chung LP, Waterer GW (2011). Genetic predisposition to respiratory infection and sepsis. Crit Rev Clin Lab Sci.

[3] Kumpf O, Schumann RR (2010). Genetic variation in innate immunity pathways and their potential contribution to the SIRS/CARS debate: evidence from human studies and animal models. J Innate Immun.

[4] Brenner D, Blaser H, Mak TW (2015). Regulation of tumour necrosis factor signalling: live or let die. Nat Rev Immunol.

[5] Barber RC, Aragaki CC, Rivera-Chavez FA, Purdue GF, Hunt JL, Horton JW (2004). TLR4 and TNF-alpha polymorphisms are associated with an increased risk for severe sepsis following burn injury. J Med Genet.

[6] Barber RC, Chang LY, Arnoldo BD, Purdue GF, Hunt JL, Horton JW, Aragaki CC (2006). Innate immunity SNPs are associated with risk for severe sepsis after burn injury. Clin Med Res.

[7] Surbatovic M, Grujic K, Cikota B, Jevtic M, Filipovic N, Romic P, Strelic N, Magic Z (2010). Polymorphisms of genes encoding tumor necrosis factor-alpha, interleukin-10, cluster of differentiation-14 and interleukin-1ra in critically ill patients. J Crit Care.

[8] Phumeetham S, Chat-Uthai N, Manavathongchai M, Viprakasit V (2012). Genetic association study of tumor necrosis factor-alpha with sepsis and septic shock in Thai pediatric patients. J Pediatr.

[9] Montoya-Ruiz C, Jaimes FA, Rugeles MT, López JÁ, Bedoya G, Velilla P (2016). Variants in LTA, TNF, IL1B and IL10 genes associated with the clinical course of sepsis. Immunol Res.

[10] Teuffel O, Ethier MC, Beyene J, Sung L (2010). Association between tumor necrosis factor-alpha promoter −308 A/G polymorphism and susceptibility to sepsis and sepsis mortality: a systematic review and meta-analysis. Crit Care Med.

[11] Allam G, Alsulaimani AA, Alzaharani AK, Nasr A (2015). Neonatal infections in Saudi Arabia: Association with cytokine gene polymorphisms. Cent Eur J Immunol.

[12] Cardoso CP, de Oliveira AJ, Botoni FA, Rezende IC, Alves-Filho JC, Cunha Fde Q, Estanislau Jde A, Magno LA, Rios-Santos F (2015). Interleukin-10 rs2227307 and CXCR2 rs1126579 polymorphisms modulate the predisposition to septic shock. Mem Inst Oswaldo Cruz.

[13] Feng B, Mao ZR, Pang K, Zhang SL, Li L (2015). Association of tumor necrosis factor α −308G/A and interleukin-6 -174G/C gene polymorphism with pneumonia-induced sepsis. J Crit Care.

[14] Gupta DL, Nagar PK, Kamal VK, Bhoi S, Rao DN (2015). Clinical relevance of single nucleotide polymorphisms within the 13 cytokine genes in North Indian trauma hemorrhagic shock patients. Scand J Trauma Resusc Emerg Med.

[15] Baghel K, Srivastava RN, Chandra A, Goel SK, Agrawal J, Kazmi HR, Raj S (2014). TNF-α, IL-6, and IL-8 cytokines and their association with TNF-α−308 G/A polymorphism and postoperative sepsis. J Gastrointest Surg.

[16] Kothari N, Bogra J, Abbas H, Kohli M, Malik A, Kothari D, Srivastava S, Singh PK (2013). Tumor necrosis factor gene polymorphism results in high TNF level in sepsis and septic shock. Cytokine.

[17] Susantitaphong P, Perianayagam MC, Tighiouart H, Liangos O, Bonventre JV, Jaber BL (2013). Tumor necrosis factor alpha promoter polymorphism and severity of acute kidney injury. Nephron Clin Pract.

[18] Azevedo ZM, Moore DB, Lima FC, Cardoso CC, Bougleux R, Matos GI, Luz RA, Xavier-Elsas P, Sampaio EP, Gaspar-Elsas MI, Moraes MO (2012). Tumor necrosis factor (TNF) and lymphotoxin-alpha (LTA) single nucleotide polymorphisms: importance in ARDS in septic pediatric critically ill patients. Hum Immunol.

[19] Song Z, Song Y, Yin J, Shen Y, Yao C, Sun Z, Jiang J, Zhu D, Zhang Y, Shen Q, Gao L, Tong C, Bai C (2012). Genetic variation in the TNF gene is associated with susceptibility to severe sepsis, but not with mortality. PLoS One.

[20] Duan ZX, Gu W, Zhang LY, Jiang DP, Zhou J, Du DY, Zen L, Chen KH, Liu Q, Jiang JX (2011). Tumor necrosis factor alpha gene polymorphism is associated with the outcome of trauma patients in Chinese Han population. J Trauma.

[21] Härtel C, Hemmelmann C, Faust K, Gebauer C, Hoehn T, Kribs A, Laux R, Nikischin W, Segerer H, Teig N, von der Wense A, Wieg C, Herting E (2011). German Neonatal Network: Tumor necrosis factor-α promoter −308 G/A polymorphism and susceptibility to sepsis in very-low-birth-weight infants. Crit Care Med.

[22] Paskulin DD, Fallavena PR, Paludo FJ, Borges TJ, Picanço JB, Dias FS, Alho CS (2011). TNF −308G > a promoter polymorphism (rs1800629) and outcome from critical illness. Braz J Infect Dis.

[23] Carregaro F, Carta A, Cordeiro JA, Lobo SM, Silva EH, Leopoldino AM (2010). Polymorphisms IL10-819 and TLR-2 are potentially associated with sepsis in Brazilian patients. Mem Inst Oswaldo Cruz.

[24] Gu W, Zeng L, Zhou J, Jiang DP, Zhang L, Du DY, Hu P, Chen K, Liu Q, Wang ZG, Jiang JX (2010). Clinical relevance of 13 cytokine gene polymorphisms in Chinese major trauma patients. Intensive Care Med.

[25] Menges T, König IR, Hossain H, Little S, Tchatalbachev S, Thierer F, Hackstein H, Franjkovic I, Colaris T, Martens F, Weismüller K, Langefeld T, Stricker J (2008). Sepsis syndrome and death in trauma patients are associated with variation in the gene encoding tumor necrosis factor. Crit Care Med.

[26] Jessen KM, Lindboe SB, Petersen AL, Eugen-Olsen J, Benfield T (2007). Common TNF-alpha, IL-1 beta, PAI-1, uPA, CD14 and TLR4 polymorphisms are not associated with disease severity or outcome from Gram negative sepsis. BMC Infect Dis.

[27] McDaniel DO, Hamilton J, Brock M, May W, Calcote L, Tee LY, Vick L, Newman DB, Vick K, Harrison S, Timberlake G, Toevs C (2007). Molecular analysis of inflammatory markers in trauma patients at risk of postinjury complications. J Trauma.

[28] Garnacho-Montero J, Aldabo-Pallas T, Garnacho-Montero C, Cayuela A, Jiménez R, Barroso S, Ortiz-Leyba C (2006). Timing of adequate antibiotic therapy is a greater determinant of outcome than are TNF and IL-10 polymorphisms in patients with sepsis. Crit Care.

[29] Schueller AC, Heep A, Kattner E, Kroll M, Wisbauer M, Sander J, Bartmann P, Stuber F (2006). Prevalence of two tumor necrosis factor gene polymorphisms in premature infants with early onset sepsis. Biol Neonate.

[30] Sipahi T, Pocan H, Akar N (2006). Effect of various genetic polymorphisms on the incidence and outcome of severe sepsis. Clin Appl Thromb Hemost.

[31] Nakada TA, Hirasawa H, Oda S, Shiga H, Matsuda K, Nakamura M, Watanabe E, Abe R, Hatano M, Tokuhisa T (2005). Influence of toll-like receptor 4, CD14, tumor necrosis factor, and interleukine-10 gene polymorphisms on clinical outcome in Japanese critically ill patients. J Surg Res.

[32] Gordon AC, Lagan AL, Aganna E, Cheung L, Peters CJ, McDermott MF, Millo JL, Welsh KI, Holloway P, Hitman GA, Piper RD, Garrard CS, Hinds CJ (2004). TNF and TNFR polymorphisms in severe sepsis and septic shock: a prospective multicentre study. Genes Immun.

[33] Jaber BL, Rao M, Guo D, Balakrishnan VS, Perianayagam MC, Freeman RB, Pereira BJ (2004). Cytokine gene promoter polymorphisms and mortality in acute renal failure. Cytokine.

[34] Balding J, Healy CM, Livingstone WJ, White B, Mynett-Johnson L, Cafferkey M, Smith OP (2003). Genomic polymorphic profiles in an Irish population with meningococcaemia: is it possible to predict severity and outcome of disease?. Genes Immun.

[35] Calvano JE, Um JY, Agnese DM, Hahm SJ, Kumar A, Coyle SM, Calvano SE, Lowry SF (2003). Influence of the TNF-alpha and TNF-beta polymorphisms upon infectious risk and outcome in surgical intensive care patients. Surg Infect (Larchmt).

[36] Schaaf BM, Boehmke F, Esnaashari H, Seitzer U, Kothe H, Maass M, Zabel P, Dalhoff K (2003). Pneumococcal septic shock is associated with the interleukin-10-1082 gene promoter polymorphism. Am J Respir Crit Care Med.

[37] Treszl A, Kocsis I, Szathmári M, Schuler A, Héninger E, Tulassay T, Vásárhelyi B (2003). Genetic Variants of TNF-α, IL-1β, IL-4 Receptor α-Chain, IL-6 and IL-10 Genes Are Not Risk Factors for Sepsis in Low-Birth-Weight Infants. Biol Neonate.

[38] Zhang D, Li J, Jiang ZW, Yu B, Tang X (2003). Association of two polymorphisms of tumor necrosis factor gene with acute severe pancreatitis. J Surg Res.

[39] Zhang DL, Li JS, Jiang ZW, Yu BJ, Tang XM, Zheng HM (2003). Association of two polymorphisms of tumor necrosis factor gene with acute biliary pancreatitis. World J Gastroenterol.

[40] Majetschak M, Obertacke U, Schade FU, Bardenheuer M, Voggenreiter G, Bloemeke B, Heesen M (2002). Tumor necrosis factor gene polymorphisms, leukocyte function, and sepsis susceptibility in blunt trauma patients. Clin Diagn Lab Immunol.

[41] Appoloni O, Dupont E, Vandercruys M, Andriens M, Duchateau J, Vincent JL (2001). Association of tumor necrosis factor-2 allele with plasma tumor necrosis factor-alpha levels and mortality from septic shock. Am J Med.

[42] Waterer GW, Quasney MW, Cantor RM, Wunderink RG (2001). Septic shock and respiratory failure in community-acquired pneumonia have different TNF polymorphism associations. Am J Respir Crit Care Med.

[43] Mira JP, Cariou A, Grall F, Delclaux C, Losser MR, Heshmati F, Cheval C, Monchi M, Teboul JL, Riché F, Leleu G, Arbibe L, Mignon A (1999). Association of TNF2, a TNF-alpha promoter polymorphism, with septic shock susceptibility and mortality: a multicenter study. JAMA.

[44] Nuntayanuwat S, Dharakul T, Chaowagul W, Songsivilai S (1999). Polymorphism in the promoter region of tumor necrosis factor-alpha gene is associated with severe meliodosis. Hum Immunol.

[45] Levy MM, Fink MP, Marshall JC, Abraham E, Angus D, Cook D, Cohen J, Opal SM, Vincent JL, Ramsay G (2003). International Sepsis Definitions Conference: 2001 SCCM/ESICM/ACCP/ATS/SIS International Sepsis Definitions Conference. Intensive Care Med.

[46] Bone RC, Balk RA, Cerra FB, Dellinger RP, Fein AM, Knaus WA, Schein RM, Sibbald WJ (1992). Definitions for sepsis and organ failure and guidelines for the use of innovative therapies in sepsis. The ACCP/SCCM Consensus Conference Committee. American College of Chest Physicians/Society of Critical Care Medicine. Chest.

[47] Kroeger KM, Steer JH, Joyce DA, Abraham LJ (2000). Effects of stimulus and cell type on the expression of the −308 tumour necrosis factor promoter polymorphism. Cytokine.

[48] Beutler B, Grau GE (1993). Tumor necrosis factor in the pathogenesis of infectious diseases. Crit Care Med.

[49] Qiu P, Cui X, Barochia A, Li Y, Natanson C, Eichacker PQ (2011). The evolving experience with therapeutic TNF inhibition in sepsis: considering the potential influence of risk of death. Expert Opin Investig Drugs.

[50] Hotchkiss RS, Moldawer LL, Opal SM, Reinhart K, Turnbull IR, Vincent JL (2016). Sepsis and septic shock. Nat Rev Dis Primers.

[51] Sweeney TE, Wong HR (2016). Risk Stratification and Prognosis in Sepsis: What Have We Learned from Microarrays?. Clin Chest Med.

[52] Sutherland AM, Russell JA (2005). Issues with polymorphism analysis in sepsis. Clin Infect Dis.

[53] Moher D, Liberati A, Tetzlaff J, Altman DG, PRISMA Group (2010). Preferred reporting items for systematic reviews and meta-analyses: the PRISMA statement. Int J Surg.

[54] Sagoo GS, Little J, Higgins JP (2009). Systematic reviews of genetic association studies. Human Genome Epidemiology Network. PLoS Med.

[55] Begg CB, Mazumdar M (1994). Operating characteristics of a rank correlation test for publication bias. Biometrics.

[56] Egger M, Davey Smith G, Schneider M, Minder C (1997). Bias in meta-analysis detected by a simple, graphical test. BMJ.

