# Supplementary material for: Tumor necrosis factor-α −308 G/A polymorphism and risk of sepsis, septic shock, and mortality: an updated meta-analysis
Source: Oncotarget. 2017 Sep 13;8(55):94910–9. doi: 10.18632/oncotarget.20862 (PMC5706923; doi:10.18632/oncotarget.20862)
Supplement: Supplementary file 1 [file oncotarget-08-94910-s001.pdf]

Tumor necrosis factor-α −308 G/A polymorphism and risk of sepsis, septic shock, and mortality: an updated meta-analysis

SUPPLEMENTARY MATERIALS

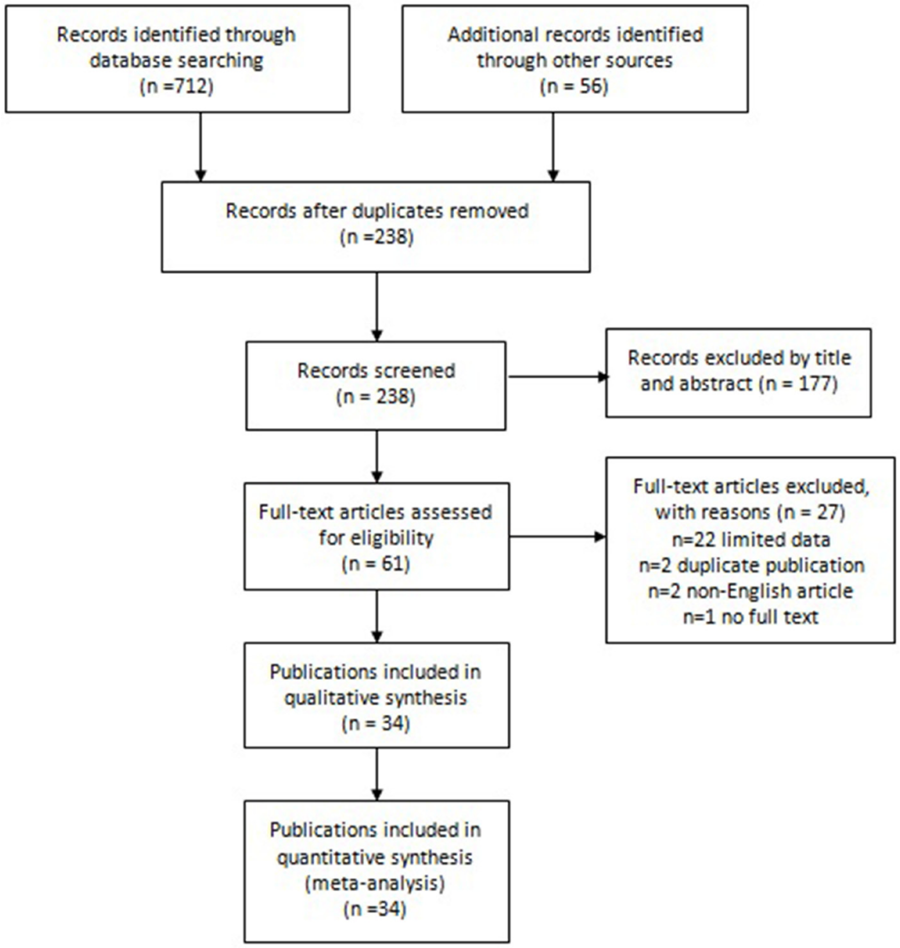

Supplementary Figure 1. Flow diagram of study identification.

Supplementary Table 1: Distribution of Tumor necrosis factor-α -308 genotype and allele among cases and controls.
See_Supplementary_Table 1

Supplementary Table 2: Quality assessment of included studies

Author/Year	HWE	Primer	Blinding	Sepsis Definition	Sepsis definition in detail
Allam et al. 2015	+	–	–	+	Defined by positive blood culture
Cardoso et al. 2015	+	–	–	+	ACCP-SCCM and International Sepsis Definitions Conference
Feng et al. 2015	+	–	–	+	International Guidelines for Management of Severe Sepsis and Septic Shock
Gupta et al. 2015	+	–	–	+	Defined by SIRS and infection
Baghel et al. 2014	–	+	–	+	ACCP-SCCM
Kothari et al. 2013	+	+	–	+	Defined by specific culture
Susantitaphong et al. 2013	–	+	+	+	International Sepsis Definitions Conference
Azevedo et al. 2012	+	–	–	+	International Pediatric Sepsis Consensus Conference
Song et al. 2012	+	+	–	+	ACCP-SCCM
Duan et al. 2011	+	+	–	+	Defined by evidence of infection and SIRS criteria
Härtel et al. 2011	+	+	–	+	Defined by positive blood culture and at least two clinical signs
Paskulin et al. 2011	+	+	+	+	ACCP-SCCM
Carregaro et al. 2010	+	–	–	+	ACCP-SCCM
Gu et al. 2010	+	–	–	+	Defined by clinical evidence of infection, body temperature and leukocyte count
Menges et al. 2008	+	+	+	+	ACCP-SCCM
Jessen et al. 2007	+	+	+	+	ACCP-SCCM
McDaniel et al. 2007	NA	+	–	+	Defined by SIRS and Positive blood culture
Garnacho-Montero et al. 2006	–	+	–	+	ACCP-SCCM
Sipahi et al. 2006	+	+	–	+	ACCP-SCCM
Schueller et al 2006	+	+	+	+	Defined by positive blood culture, leukocytes count, CRP, and clinic sign
Nakada et al. 2005	+	+	–	+	ACCP-SCCM
Gordon et al. 2004	+	+	+	+	ACCP-SCCM
Jaber et al. 2004	NA	+	+	+	NA
Balding et al. 2003	+	+	–	–	-
Calvano et al. 2003	+	+	+	+	Defined by SIRS and positive cultures
Schaaf et al. 2003	+	+	–	+	ACCP-SCCM
Treszl et al. 2003	+	+	–	+	Defined by symptom, laboratory finding and blood culture
Zhang et al. 2003	–	+	–	+	ACCP-SCCM
Zhang et al. 2003	–	+	–	+	ACCP-SCCM
Majetschak et al. 2002	+	+	–	+	ACCP-SCCM
Appoloni et al. 2001	NA	–	–	–	-
Waterer et al. 2001	–	+	+	+	ACCP-SCCM
Mira et al. 1999	NA	+	+	+	ACCP-SCCM
Nuntayanuwat et al. 1999	+	+	–	+	Defined by positive blood culture

+ Yes; – No; ACCP: American College of Chest Physicians; NA: Not available; SCCM: Society of Critical Care Medicine.
