# Supplementary material for: Tumor necrosis factor-α −308 G/A polymorphism and risk of sepsis, septic shock, and mortality: an updated meta-analysis
Source: Oncotarget. 2017 Sep 13;8(55):94910–9. doi: 10.18632/oncotarget.20862 (PMC5706923; doi:10.18632/oncotarget.20862)
Supplement: Supplementary file 2 [file oncotarget-08-94910-s002.docx]

**Supplementary Table 1. Distribution of Tumor necrosis factor-α -308 genotype and allele among cases and controls**

| Author | Year | Country | Ethnicity | Cases | Controls | Cases | | | Controls | | |
| --- | --- | --- | --- | --- | --- | --- | --- | --- | --- | --- | --- |
|  |  |  |  |  |  | GG | GA | AA | GG | GA | AA |
| **Overall sepsis** |  |  |  |  |  |  |  |  |  |  |  |
| Allam et al | 2015 | Saudi Arabia | Asian | 69 | 68 | 45 | 19 | 5 | 11 | 30 | 27 |
| Gupta et al | 2015 | India | Asian | 25 | 89 | 22 | 1 | 2 | 69 | 17 | 3 |
| Baghel et al | 2014 | India | Asian | 47 | 192 | 24 | 15 | 8 | 140 | 43 | 9 |
| Kothari et al | 2013 | India | Asian | 169 | 224 | 95 | 61 | 13 | 160 | 57 | 7 |
| Susantitaphong et al | 2013 | USA | Mixed | 112 | 150 | 76 | 30 | 6 | 106 | 36 | 8 |
| Azevedo et al | 2012 | Brazil | Mixed | 439 | 564 | 348 | 80 | 11 | 448 | 105 | 11 |
| Song et al | 2012 | China | Asian | 802 | 600 | 721 | 79 | 2 | 560 | 38 | 2 |
| Duan et al | 2011 | China | Asian | 131 | 174 | 86 | 39 | 6 | 138 | 33 | 3 |
| Härtel et al 1 | 2011 | Germany | Mixed | 354 | 1590 | 270 | 80 | 4 | 1127 | 427 | 36 |
| Härtel et al 2 | 2011 | Germany | Mixed | 149 | 777 | 107 | 36 | 6 | 562 | 200 | 15 |
| Paskulin et al | 2011 | Brazil | Caucasian | 349 | 171 | 245 | 99 | 5 | 129 | 42 | 0 |
| Carregaro et al | 2010 | Brazil | Mixed | 97 | 206 | 74 | 23 | 0 | 146 | 57 | 3 |
| Gu et al | 2010 | China | Asian | 131 | 174 | 86 | 39 | 6 | 138 | 33 | 3 |
| Menges et al 1 | 2008 | Germany | Caucasian | 71 | 83 | 38 | 33 | | 74 | 9 | |
| Menges et al 2 | 2008 | Germany | Caucasian | 17 | 59 | 8 | 9 | | 44 | 15 | |
| McDaniel et al 1 | 2007 | USA | African American | 16 | 16 | 11 | 5 | | 12 | 4 | |
| McDaniel et al 2 | 2007 | USA | Caucasian | 15 | 21 | 11 | 4 | | 17 | 4 | |
| Garnacho-Montero et al | 2006 | Spain | Caucasian | 224 | 101 | 186 | 35 | 3 | 82 | 15 | 4 |
| Schueller et al | 2006 | Germany | Caucasian | 67 | 102 | 48 | 16 | 3 | 68 | 29 | 5 |
| Sipahi et al | 2006 | Turkey | Caucasian | 53 | 77 | 42 | 11 | 0 | 70 | 7 | 0 |
| Nakada et al | 2005 | Japan | Asian | 86 | 325 | 81 | 5 | 0 | 319 | 6 | 0 |
| Gordon et al | 2004 | UK and Australia | Caucasian | 212 | 354 | 135 | 69 | 8 | 233 | 110 | 11 |
| Jaber et al | 2004 | USA | Caucasian | 40 | 21 | 21 | 19 | | 12 | 9 | |
| Balding et al | 2003 | Ireland | Caucasian | 183 | 389 | 100 | 75 | 8 | 233 | 140 | 16 |
| Calvano et al | 2003 | Spain | Mixed | 23 | 21 | 20 | 2 | 1 | 18 | 0 | 3 |
| Schaaf et al | 2003 | Germany | Caucasian | 50 | 68 | 32 | 16 | 2 | 49 | 15 | 4 |
| Treszl et al | 2003 | Hungary | Caucasian | 33 | 70 | 25 | 8 | 0 | 57 | 13 | 0 |
| Zhang et al | 2003 | China | Asian | 32 | 116 | 15 | 17 | | 82 | 26 | 8 |
| Zhang et al | 2003 | China | Asian | 18 | 102 | 9 | 9 | | 76 | 21 | 5 |
| Majetschak et al | 2002 | Netherlands | Caucasian | 14 | 56 | 10 | 4 | 0 | 36 | 20 | 0 |
| Waterer et al | 2001 | Australia | Mixed | 31 | 249 | 24 | 4 | 3 | 173 | 60 | 16 |
| Mira et al | 1999 | France | Caucasian | 89 | 87 | 54 | 35 | | 71 | 16 | |
| Nuntayanuwat et al | 1999 | Thailand | Asian | 72 | 74 | 46 | 25 | 1 | 60 | 14 | 0 |
| **Septic shock** |  |  |  |  |  |  |  |  |  |  |  |
| Kothari et al | 2013 | India | Asian | 72 | 224 | 47 | 18 | 7 | 160 | 57 | 7 |
| Paskulin et al | 2011 | Brazil | Caucasian | 248 | 171 | 180 | 63 | 5 | 129 | 42 | 0 |
| Garnacho-Montero et al | 2006 | Spain | Caucasian | 114 | 101 | 92 | 22 | | 82 | 15 | 4 |
| Calvano et al | 2003 | Caucasian | Mixed | 5 | 21 | 5 | 0 | 0 | 18 | 0 | 3 |
| Schaaf et al | 2003 | Germany | Caucasian | 12 | 68 | 8 | 3 | 1 | 49 | 15 | 4 |
| Zhang et al | 2003 | China | Asian | 32 | 116 | 15 | 17 | | 82 | 26 | 8 |
| Zhang et al | 2003 | China | Asian | 18 | 102 | 9 | 9 | | 76 | 21 | 5 |
| Mira et al | 1999 | France | Caucasian | 89 | 87 | 54 | 35 | | 71 | 16 | |
| **Sepsis mortality** |  |  |  |  |  |  |  |  |  |  |  |
| Cardoso et al | 2015 | Brazil | Mixed | 13 | 56 | 10 | 2 | 1 | 44 | 12 | 0 |
| Feng et al | 2015 | China | Asian | 63 | 214 | 59 | 3 | 1 | 202 | 12 | 0 |
| Azevedo et al | 2012 | Brazil | Mixed | 40 | 399 | 36 | 2 | 2 | 312 | 78 | 9 |
| Song et al | 2012 | China | Asian | 154 | 273 | 137 | 17 | 0 | 232 | 39 | 2 |
| Paskulin et al | 2011 | Brazil | Caucasian | 195 | 154 | 137 | 58 | | 108 | 46 | |
| Jessen et al | 2007 | Denmark | Caucasian | 60 | 244 | 34 | 20 | 6 | 148 | 86 | 10 |
| Garnacho-Montero et al | 2006 | Spain | Caucasian | 52 | 172 | 45 | 7 | | 141 | 31 | |
| Sipahi et al | 2006 | Turkey | Caucasian | 19 | 34 | 15 | 4 | 0 | 27 | 7 | 0 |
| Nakada et al | 2005 | Japan | Asian | 22 | 64 | 18 | 4 | 0 | 63 | 1 | 0 |
| Gordon et al | 2004 | UK and Australia | Caucasian | 52 | 160 | 39 | 12 | 1 | 96 | 57 | 7 |
| Balding et al | 2003 | Ireland | Caucasian | 25 | 158 | 14 | 10 | 1 | 85 | 66 | 7 |
| Calvano | 2003 | Spain | Mixed | 8 | 15 | 8 | 0 | 0 | 12 | 2 | 1 |
| Schaaf et al | 2003 | Germany | Caucasian | 5 | 45 | 4 | 0 | 1 | 28 | 16 | 1 |
| Appoloni et al | 2001 | Belgium | Caucasian | 15 | 19 | 8 | 7 | | 17 | 2 | |
| Waterer et al | 2001 | Australia | Mixed | 15 | 16 | 12 | 1 | 2 | 12 | 3 | 1 |
| Mira et al | 1999 | France | Caucasian | 48 | 41 | 23 | 25 | | 31 | 10 | |
